# Autologous Fat Grafting—A Panacea for Scar Tissue Therapy?

**DOI:** 10.3390/cells13161384

**Published:** 2024-08-20

**Authors:** Nura Ahmad, Alexandra Anker, Silvan Klein, Jillian Dean, Leonard Knoedler, Katya Remy, Andrea Pagani, Sally Kempa, Amraj Terhaag, Lukas Prantl

**Affiliations:** 1 Department of Plastic, Hand and Reconstructive Surgery, University Hospital Regensburg, Franz–Josef–Strauß Allee 11, 93053 Regensburg, Germany; alexandra.anker@ukr.de (A.A.); silvan.klein@ukr.de (S.K.); lknoedler@mgh.harvard.edu (L.K.); andrea.pagani@klinik.uni-regensburg.de (A.P.); sally.kempa@klinik.uni-regensburg.de (S.K.); amraj.terhaag@klinik.uni-regensburg.de (A.T.); lukas.prantl@ukr.de (L.P.); 2 School of Medicine, University of Pittsburgh, Pittsburgh, PA 15261, USA; jid99@pitt.edu; 3 Division of Plastic and Reconstructive Surgery, Massachusetts General Hospital, Harvard Medical School, Boston, MA 02114, USA; kremy11@mgh.harvard.edu

**Keywords:** autologous fat grafting, scar therapy, adipose-derived stem cells, tissue regeneration

## Abstract

Scars may represent more than a cosmetic concern for patients; they may impose functional limitations and are frequently associated with the sensation of itching or pain, thus impacting both psychological and physical well-being. From an aesthetic perspective, scars display variances in color, thickness, texture, contour, and their homogeneity, while the functional aspect encompasses considerations of functionality, pliability, and sensory perception. Scars located in critical anatomic areas have the potential to induce profound impairments, including contracture-related mobility restrictions, thereby significantly impacting daily functioning and the quality of life. Conventional approaches to scar management may suffice to a certain extent, yet there are cases where tailored interventions are warranted. Autologous fat grafting emerges as a promising therapeutic avenue in such instances. Fundamental mechanisms underlying scar formation include chronic inflammation, fibrogenesis and dysregulated wound healing, among other contributing factors. These mechanisms can potentially be alleviated through the application of adipose-derived stem cells, which represent the principal cellular component utilized in the process of lipofilling. Adipose-derived stem cells possess the capacity to secrete proangiogenic factors such as fibroblast growth factor, vascular endothelial growth factor and hepatocyte growth factor, as well as neurotrophic factors, such as brain-derived neurotrophic factors. Moreover, they exhibit multipotency, remodel the extracellular matrix, act in a paracrine manner, and exert immunomodulatory effects through cytokine secretion. These molecular processes contribute to neoangiogenesis, the alleviation of chronic inflammation, and the promotion of a conducive milieu for wound healing. Beyond the obvious benefit in restoring volume, the adipose-derived stem cells and their regenerative capacities facilitate a reduction in pain, pruritus, and fibrosis. This review elucidates the regenerative potential of autologous fat grafting and its beneficial and promising effects on both functional and aesthetic outcomes when applied to scar tissue.

## 1. Introduction

In recent years, the field of regenerative medicine has made significant strides toward developing treatments that address the complex challenges of scar tissue management. Among these advancements, autologous fat grafting (AFG) has gained prominence as a transformative approach, offering a dual-action solution that extends beyond superficial cosmetic enhancement to address the functional impairments often associated with scarred tissues [1]. Initially conceptualized for the aesthetic improvement of depressed facial scars, the methodology behind AFG involves the strategic harvesting and transplantation of adipose tissue from one part of the body to another [2]. This approach has evolved substantially from its origins, now supported by an extensive body of scientific research that delineates its volumetric and regenerative benefits.

Central to the success of AFG is the critical role played by adipose-derived stem cells (ADSCs), which are found in abundance within adipose tissue. These cells are key drivers of the wound healing process, contributing significantly to dermal scar remediation. The application of ADSCs through AFG not only promises aesthetic enhancements but also provides therapeutic benefits, including the alleviation of pain and itchiness, restoration of volume, and the promotion of functional recovery in scarred tissues [3].

The exploration of AFG and its integration with ADSCs underscores a pivotal shift towards harnessing the body’s intrinsic regenerative capabilities to address scar management [1]. The interplay between the volumetric enhancement provided by the transferred fat and the regenerative functions of ADSCs has opened new avenues for comprehensive scar treatment. ADSCs, with their potent regenerative properties, including the secretion of angiogenic, anti-inflammatory, and immunomodulatory factors, act synergistically with the physical benefits of fat grafting to facilitate not only the cosmetic improvement of scars but also their structural and functional healing [4]. This synergy points to a broader implication of AFG in regenerative medicine, suggesting its potential applicability, beyond scar treatment, to the healing of wounds, the repair of tissue damages from various causes, and the improvement of other dermatological conditions. By expanding our understanding of the biological actions of ADSCs within the context of AFG, this paper aims to provide a comprehensive overview of the current state of research in the field of AFG for scar treatment, comprising the mechanisms through which AFG and ADSCs exert their effects on scar tissue, offering insight into the potential of these treatments to revolutionize scar management strategies within the broader scope of tissue regeneration. Thus, a comprehensive review of the literature was performed.

## 2. Autologous Fat Grafting as a New Approach to Scar Treatment with Regenerative Potential

In 1893, Neuber, A. pioneered the use of AFG for the amelioration of scars, marking a significant advancement in the treatment of depressed facial scars caused by trauma. This approach entailed the extraction of a minimal quantity of adipose tissue from the forearm, which was then transplanted into the affected scar areas. The intervention was noted for its efficacy, with reports indicating positive outcomes [5]. Over recent years, this technique has garnered increased interest and recognition due to its dual beneficial effects of volumetric enhancement and regenerative capabilities. These regenerative capabilities of AFG on scarred tissue are largely attributed to the presence of ADSCs within the fat grafts [6]. ADSCs are abundantly found in adipose tissue and have been evidenced, through both clinical and animal research, to play a pivotal role in enhancing various aspects of the wound healing process, especially in the context of dermal scars [7]. AFG has demonstrated efficacy in improving the aesthetic appearance of scars, while also reducing pain and pruritus, among other advantageous effects on scar-related conditions, which will be further elaborated upon [8].

### Properties of Adipose-Derived Stem Cells and Their Potential Effects on Scars

ADSCs have become a central element in the field of regenerative medicine due to their significant therapeutic potential for repairing scars and similar conditions, leading to an increased preference for using lipofilling as a primary method for treating various types of scars [9,10]. Recent investigations reveal that ADSCs, present within the stromal vascular fraction (SVF) of adipose tissue, possess proangiogenic properties akin to those of bone marrow-derived stem cells [11,12,13]. These studies, including in vivo experiments with murine models, have highlighted that ADSCs not only secrete angiogenic and antiapoptotic factors [14], but also exhibit the capability to differentiate into endothelial cells and incorporate into existing vascular networks [15]. Such activities promote neovascularization in ischemic tissues. Furthermore, ADSCs exert immunomodulatory effects through the secretion of cytokines and have the potential to differentiate into various cell lineages, including adipogenic, chondrogenic, osteogenic, neural, myogenic, and endothelial cells [10,14,16].

Within the ambit of regenerative medicine and specifically AFG for scar treatment, the role of ADSCs extends into a complex network of biological pathways, signaling axes, and molecular interactions that collectively enhance tissue repair and regeneration. The involvement of the TLR4/NF-κB signaling pathway in ADSC-mediated anti-inflammatory responses presents a noteworthy mechanism, through which ADSCs exert their immunomodulatory effects, mitigating inflammation and thus facilitating an environment conducive to healing and tissue regeneration [17]. Upon activation, NF-κB translocates to the nucleus, upregulating the expression of pro-inflammatory genes [18]. However, ADSCs can attenuate this pathway, leading to a reduction in inflammatory cytokines, such as TNF-α and IL-6, thus mitigating inflammation and promoting a conducive healing environment [19]. This pathway underscores the ADSCs’ ability to modulate immune responses, which is critical in the context of scar treatment where chronic inflammation can perpetuate fibrosis and impair healing. Beyond the TLR4/NF-κB signaling pathway, ADSCs are also known to interact with the STAT3 pathway, a critical mediator in the resolution of inflammation and promotion of tissue repair [20]. Activation of STAT3 leads to the transcription of anti-inflammatory genes [21], further contributing to the reduction in the pro-inflammatory milieu in scarred tissues [22,23,24].

The proangiogenic attributes of ADSCs have been especially significant in treating degenerative and chronic lesions characterized by low perfusion, such as those resulting from oncologic radiation treatments. Dramatic symptomatic improvements have been documented following autologous fat grafting, which can be partially attributed to the action of ADSCs [16,25]. For instance, Rigotti et al.‘s research demonstrated that the application of ADSCs effectively mitigated severe symptoms following radiation therapy, including atrophy, retraction, fibrosis, and ulcer formation [25]. Notably, histological analyses post-ADSC-enriched fat grafting have shown skin structures that more closely resemble those of non-scarred tissue, evidenced by organized collagen deposition, enhanced vascularization, and the regeneration of the papillary dermis [26,27].

ADSCs influence the wound healing process and scar tissue remodeling through several mechanisms:

Angiogenesis: By secreting proangiogenic factors such as vascular endothelial growth factor (VEGF), fibroblast growth factor (FGF), and hepatocyte growth factor (HGF), ADSCs stimulate the formation of new blood vessels, enhancing tissue perfusion and oxygenation [28]. VEGF, for instance, acts by binding to its receptors (VEGFRs) on the surface of endothelial cells, triggering a cascade of intracellular events, including the activation of the PI3K/Akt pathway, leading to endothelial cell proliferation and migration [28,29,30,31,32,33,34]. FGF contributes to the proliferation and differentiation of endothelial cells, while HGF promotes angiogenesis and tissue regeneration. The combined activity of these factors culminates in the formation of new capillary structures, which are crucial for the revascularization and subsequent healing of ischemic and scarred tissues [35].

The interplay between ADSCs and the HIF-1α/VEGF axis emerges as a pivotal mechanism by which ADSCs promote vascularization within scarred tissues. The hypoxic environment of damaged tissues activates HIF-1α, a transcription factor that upregulates the expression of VEGF, thereby enhancing angiogenesis and improving tissue perfusion and oxygenation [36]. This process is crucial for delivering nutrients and oxygen to the healing tissue, promoting the survival of grafted fat cells and the regeneration of the scarred area [37]. Additionally, Sphingosine-1-phosphate (S1P), a bioactive lipid mediator secreted by ADSCs, has emerged as a significant player in promoting angiogenesis, enhancing endothelial cell migration and stabilizing new blood vessels. Its role underscores the complexity of the angiogenic process essential for supplying nutrients and oxygen to healing tissues [32].

While VEGF, FGF, and HGF are central to the angiogenic process, Angiopoietin-1 (Ang-1) and EphrinB2/EphB4 also merit attention for their roles in vascular stabilization and maturation [38]. Ang-1, acting through the Tie2 receptor, plays a pivotal role in stabilizing and maturing newly formed blood vessels, complementing the actions of VEGF [39]. The EphrinB2/EphB4 signaling axis is crucial for arteriovenous differentiation, further detailing the molecular intricacies of angiogenesis facilitated by ADSCs in the context of AFG [34].

Immunomodulation: ADSCs modulate the immune response by secreting anti-inflammatory cytokines, which can alter the inflammatory phase of wound healing, potentially reducing scar formation [36,40,41,42,43,44,45,46]. The immunomodulatory function of ADSCs is largely attributed to their production of cytokines such as IL-10 and TGF-β. IL-10 is known for its anti-inflammatory effects, including the downregulation of pro-inflammatory cytokine synthesis and the inhibition of antigen-presenting cells [47]. TGF-β has a broader role, including the modulation of immune cell proliferation, differentiation, and survival, as well as the conversion of effector T-cells into T_regs_, which play a crucial role in maintaining immune tolerance [41]. Through these pathways, ADSCs can attenuate the inflammatory environment. Beyond IL-10 and TGF-β, ADSCs produce a range of other cytokines and chemokines, which are crucial for modulating the healing environment. Interleukin-6 (IL-6), while often considered pro-inflammatory, can have regenerative roles, depending on the context, contributing to the transition from inflammation to healing [42]. Similarly, interleukin-4 (IL-4) promotes anti-inflammatory responses and assists in tissue remodeling by influencing macrophage polarization towards an M2 phenotype, which is associated with tissue repair and regeneration [43]. The JAK/STAT-signaling pathway is another critical route through which ADSCs promote cellular communication and immunomodulation. The activation of this pathway can lead to the transcription of genes involved in cell growth, survival, and differentiation, which are essential for effective wound healing [48].

Cell differentiation: The ability of ADSCs to differentiate into multiple cell types is pivotal for tissue repair. Through the regulation of signaling pathways such as the Wnt/β-catenin pathway for osteogenic differentiation [49] or PPARγ activation for adipogenic differentiation [50], ADSCs replace damaged cells and contribute to the restoration of tissue architecture [51]. This pluripotency allows for the repopulation of diverse cell types, aiding in the functional recovery of the damaged tissue, whether it be skin, bone, cartilage, or vascular structures [51].

**Figure 1:**
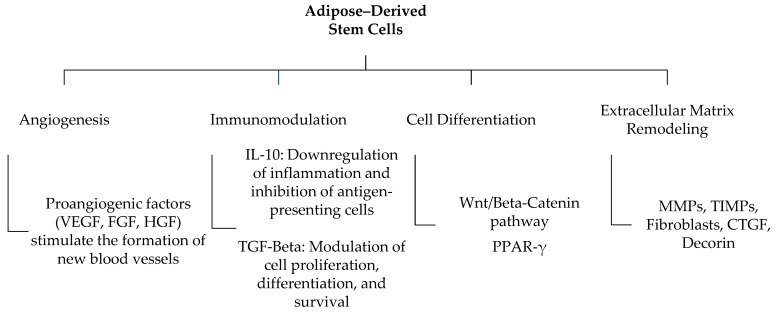
Adipose-derived stem cells: properties and potential effects on scars. Angiogenesis: ADSCs secrete proangiogenic factors (VEGF, FGF, HGF) that promote new blood vessel formation, improving tissue oxygenation and perfusion. They also interact with the HIF-1α/VEGF axis and produce Sphingosine-1-phosphate (S1P) for vessel stabilization; immunomodulation: ADSCs release anti-inflammatory cytokines (IL-10, TGF-β) that reduce inflammation and scar formation. They also secrete other cytokines (IL-6, IL-4) and activate the JAK/STAT pathway to support healing; cell differentiation: ADSCs differentiate into various cell types, aiding tissue repair and regeneration through pathways such as Wnt/β-catenin and PPARγ. Extracellular matrix remodeling: ADSCs modulate collagen and matrix components, enhancing structural integrity and reducing fibrosis. They secrete MMPs for matrix breakdown and TIMPs to balance synthesis and degradation, influencing fibroblasts and secreting CTGF and Decorin for organized ECM formation.

Extracellular matrix remodeling: ADSCs influence the deposition and organization of collagen and other matrix components, contributing to the structural integrity and functional recovery of scarred tissue. ECM remodeling is a critical component of scar maturation and resolution [52,53,54,55,56]. ADSCs secrete various MMPs that break down over accumulated ECM proteins, allowing for the proper reorganization of the matrix [53]. Concurrently, the secretion of TIMPs regulates this breakdown, ensuring a balance between ECM synthesis and degradation [54,57]. Additionally, ADSCs can influence fibroblasts within the scar tissue to modulate their collagen production, promoting a more organized and less fibrotic ECM that resembles that of normal, healthy tissue [58]. Connective tissue growth factor (CTGF) and Decorin are additional molecules worth discussing. CTGF is a key mediator in connective tissue remodeling, working with TGF-β to stimulate fibroblast proliferation, collagen synthesis, and tissue repair [55]. Decorin, a small leucine-rich proteoglycan, plays a crucial role in regulating collagen fibrillogenesis and inhibiting TGF-β activity, thus preventing excessive fibrosis [56]. These molecules, secreted by ADSCs, further detail the complex regulation of extracellular matrix remodeling in scar healing, providing a more nuanced understanding of how AFG influences tissue structure and function. By engaging in these pathways, ADSCs offer a multifaceted approach to tissue regeneration, underscoring their utility in enhancing the healing of scar tissue and improving the outcomes of lipofilling procedures (Figure 1).

Overall, ADSCs facilitate comprehensive tissue regeneration and improve healing outcomes in scarred tissues.

## 3. Techniques and Clinical Implication of Autologous Fat Grafting

Autologous adipose tissue, recognized for its accessibility, availability, high biocompatibility, and minimal surgical trauma, has become a cornerstone in clinical practices for both defect repair and aesthetic enhancements (Table 1). The evolution of fat grafting techniques, categorized by adipose particle size—macrofat, microfat, and nanofat—each serves distinct clinical purposes, tailored to their specific application methodologies [59]. Macrofat grafting utilizes particles larger than 2.4 mm, making it ideal for substantial volume augmentation in areas such as the breasts and buttocks. The use of blunt needles with a diameter of 2 mm for injection is necessitated by this approach [59]. Transitioning to microfat, which requires a cannula with a hole diameter ranging from 1.2 to 2.4 mm, is optimized for augmenting more delicate areas such as the forehead, eyelids, and hands. The smaller particle size not only reduces the risks associated with larger fat particles but also facilitates a smoother injection process, making it well-suited for detailed and nuanced enhancements [59]. The innovation in fat grafting is exemplified by the introduction of nanofat, which involves the mechanical emulsification and filtration of microfat. The composition of the harvested adipose tissue is also a critical success factor in fat grafting. Adipose tissue comprises mature adipocytes and a stromal vascular fraction (SVF), rich in various cellular components including ADSCs, endothelial cells, pericytes, and immune cells. These components are vital for the integration and survival of the graft [60]. This process yields a mixture with two cell types: stromal vascular fraction (SVF) cells and ADSCs. In seminal research by Tonnard and colleagues, it was discovered that every 100 mL of nanofat contains between 1.9 to 3 million active SVF-derived stem cells, along with 100,000 to 200,000 CD34+ cells [61]. Remarkably, these concentrations are consistent regardless of the initial adipose tissue processing method, illustrating the robustness of nanofat in producing a cellular mixture vital for tissue repair and regeneration. The SVF and CD34+ cells in nanofat have the capacity to transform into mature adipocytes, similar in quality and quantity to those found in macrofat and microfat. CD34+ cells exhibit significant proliferative capabilities and are more adept at colony formation [62]. Furthermore, CD34+ cells within adipose tissue share several properties with ADSCs, which are crucial for tissue maintenance and remodeling [63]. Additionally, it has been demonstrated that MSCs enhance the viability of fat grafts, primarily through the promotion of angiogenesis [64,65]. Leveraging the regenerative properties of SVF cells and ADSCs, nanofat has shown promising outcomes in improving skin quality and ameliorating scars, achieving high patient satisfaction, with minimal adverse effects.

The centrifugation process for isolating SVF plays a critical role in ensuring the optimal separation of cells from adipose tissue, which is pivotal for the regenerative potential of nanofat grafting. Baptista and colleagues isolated SVF by lysing red blood cells (RBCs) in lipoaspirate, followed by centrifugation at 900× *g* for 15 min, and then resuspending the SVF-rich pellet. They reported obtaining an average of 24.0 ± 7.4 × 10^4^ cells per milliliter, with 1.2 ± 0.37 × 10^4^ of these cells exhibiting plastic adherence. The adherent cells were characterized by the absence and presence of specific markers: CD45−, CD73−, CD31+, CD44+, CD90+, CD105+, and CD34+. This technique was contrasted with a manual enzymatic method, which yielded higher numbers of both total and plastic-adherent cells (58.4 ± 17.8 × 10^4^ and 8.5 ± 6.7 × 10^4^ cells per milliliter, respectively), but required more time. The cryopreservation of cells at −196 °C was noted to decrease cell yield [66]. Following Baptista et al., Condé-Green and colleagues applied the same mechanical isolation process and observed a higher quantity of MSCs and endothelial cells in the pellet from centrifuged lipoaspirates compared to decanted ones, though without comparison to enzymatic methods. They also compared two mechanical methods: centrifugation and vortexing, followed by centrifugation, finding that centrifugation doubled the cell yield compared to vortexing, with both methods maintaining high cell viability. Comparatively, collagenase digestion resulted in a tenfold increase in SVC yield but with a higher proportion of hematopoietic cells and fewer ASCs and endothelial cells [67].

Markarian and colleagues introduced three modifications to Baptista’s mechanical isolation method. Their first protocol involved RBC lysis, followed by a 600× *g* centrifugation for 10 min. The next two methods added an extra centrifugation step at 800× *g* and 1280× *g*, respectively, each for 15 min, before repeating the 600× *g* centrifugation. The cells isolated by these methods were viable, yet proliferation ceased after 14 days. These techniques were compared with the collagenase and trypsin isolation methods, with collagenase showing significantly greater cell yields than trypsin or the mechanical methods. No growth in cell cultures limited further analysis of the mechanically isolated SVFs [68].

Shah et al. isolated SVF through repeated washing with PBS, vigorous hand shaking, and centrifugation at 1200 rpm for 5 min. This method, compared to collagenase-based isolation, resulted in an increase in CD45+ cells and showed significant differences in other cell markers, indicating changes in the cell phenotype. The mechanically isolated cells, requiring only a third of the time needed for enzymatic methods, yielded significantly fewer cells but demonstrated comparable differentiation potential [69]. On the other hand, Romanov et al. diluted lipoaspirate, vortexed it for 2 to 3 min, and then centrifuged it at 600× *g* for 10 min. The cultured cells consistently expressed α-smooth actin and produced type 1 collagen and fibronectin. They displayed endothelial characteristics and differentiated into adipogenic, osteogenic, and neurogenic lineages, although no comparisons with enzymatic isolation were made [70]. Raposio et al. reported isolating SVF in an operating room by submitting lipoaspirate to 6000 vibrations per minute for 6 min, followed by centrifugation at 1600 rpm for 6 min. This method yielded an average of 1 × 10^7^ SVCs from 80 mL of lipoaspirate, 5% of which were ASCs, but lacked extensive in vitro characterization or in vivo outcome measurements, limiting conclusions on clinical benefits [71].

Moreover, the choice of donor site has been a topic of debate, with studies showing that while there are no significant differences in fat quality or graft survival among common sites, such as the abdomen, buttocks, thighs, and knees, the specific choice often depends on patient-specific factors such as body type and the amount of accessible fat [72]. This suggests a tailored approach, where the selection of the donor site should consider both the ease of access and the specific clinical needs of the patient, enhancing the overall efficacy of the graft [73]. Tonnard et al.‘s finding suggests that nanofat may offer superior regenerative properties, making it particularly useful for therapeutic applications such as scar revision and chronic wound treatment. Studies on macrofat and microfat have pointed out limitations primarily associated with the size of the adipose particles used in these techniques [61]. While macrofat is effective for creating substantial volume, its larger particles pose a higher risk of embolism, particularly when used in superficial tissues. Microfat, with its smaller particle size, offers safer and more precise enhancements for delicate areas such as the eyelids and hands, emphasizing its suitability for detailed cosmetic improvements [59,74,75].

Further comparative studies have focused on the outcomes based on the harvesting and processing techniques. The conventional methods of liposuction, often characterized by higher vacuum pressures, have been shown to induce significant trauma to adipose tissue of up to 90%, leading to decreased cell viability and increased graft loss [73]. This contrasts sharply with the tumescent technique, where a large volume of a dilute solution of local anesthetic and epinephrine is injected into the donor site prior to fat removal, minimizing tissue trauma and blood loss, thereby preserving the structural integrity of the adipose tissue [76].

The evolution of fat grafting techniques from macrofat to nanofat represents a significant shift towards utilizing adipose tissue not only for aesthetic enhancements but also for its regenerative potential. Each technique, with its particular method of harvesting and processing, offers distinct benefits and limitations, necessitating careful consideration of the patient’s specific needs and the intended clinical outcomes. As the field advances, ongoing research is essential to further refine these techniques, standardize protocols, and optimize outcomes, ensuring that autologous fat grafting remains a vital tool in both cosmetic and reconstructive surgery.

### 3.1. Effects of Autologous Fat Grafting on Scar

AFG utilizes the unique properties of ADSCs to enhance scar appearance. This is achieved as ADSCs facilitate the integration of a newly deposited adipocyte matrix with the existing scar tissue, which serves to smooth out textural anomalies and mitigate pigmentation variances [77]. The release of growth factors and cytokines by ADSCs, such as VEGF, HGF, and FGF, catalyzes tissue repair and regeneration [9,78,79,80,81,82,83,84,85,86,87].

The alleviation of itchiness that often accompanies scar tissue is another therapeutic benefit of AFG. Pruritus, or itching, in scarred areas typically arises from a confluence of disrupted skin integrity and localized inflammatory processes [6]. ADSCs address these factors through the modulation of cytokine profiles, dampening the pro-inflammatory signals that exacerbate itching [80]. Central to this process is the regulation of interleukin-31 (IL-31), a cytokine associated with the sensation of itch. ADSCs have the ability to alter the expression or activity of IL-31, among other pruritogenic mediators, contributing to the reduction of pruritus [88]. Additionally, ADSCs may influence the restoration of the skin barrier function, which is often compromised in scarred tissue. By improving the barrier, and thus the microenvironment of the skin, the pathological stimuli that induce pruritus are lessened [83].

Moreover, ADSCs contribute to the modulation of the wound healing process through the secretion of extracellular vesicles (EVs) [89] that contain a wide array of bioactive molecules, including microRNAs (miRNAs), growth factors, and cytokines, such as, TGF-β, VEGF, FGF, PDGF, and IL-10, and chemokines, CCL2 (MCP-1) and CXCL12 (SDF-1) [84,90,91,92]. Chemokines such as CXCL12 (SDF-1) are central in recruiting stem cells and other reparative cells to the site of injury, facilitating the repair process [86]. CCL5 (RANTES), another chemokine secreted by ADSCs, helps in recruiting immune cells but also plays a role in angiogenesis and cell proliferation, contributing to the complex process of wound healing [87,93]. These EVs play a significant role in cell-to-cell communication, influencing the behavior of surrounding cells and thereby promoting tissue repair and regeneration [94]. The delivery of these miRNAs and proteins can alter gene expression in recipient cells, facilitating processes such as cell proliferation, differentiation, and extracellular matrix remodeling, which are essential for scar improvement and healing [95]

The ADSCs’ secretome, which is rich in anti-inflammatory and immunomodulatory factors, plays a crucial role in this remediation. By secreting molecules that encourage a shift from a pro-inflammatory to an anti-inflammatory state, ADSCs can change the local environment to one that favors healing, reducing the chronic inflammation that often leads to persistent itchiness [96,97,98]. Beyond cytokine modulation, ADSCs also contribute to the repair of damaged nerve endings that may be involved in the itch response. Through the release of neurotrophic factors, ADSCs support the regeneration of neural pathways, which can help normalize the signaling mechanisms that communicate the sensation of itch to the brain [99]. ADSCs also have the ability to modulate the senescence-associated secretory phenotype (SASP) of the senescent cells within scar tissue. They secrete factors that can reduce the expression of SASP components such as IL-1β, IL-6, and MMPs, which are known to contribute to inflammation and matrix degradation. By altering the SASP, ADSCs help to reduce chronic inflammation and promote a regenerative environment conducive to scar healing [90,100]. The complex interplay among angiogenic, anti-inflammatory, immunomodulatory, and neurotrophic factors released by ADSCs positions AFG as a potent approach for comprehensive scar management. This strategy promotes not just cosmetic repair but also functional healing, potentially transforming the quality of life for individuals bearing the physical and psychological burdens of scarring.

### 3.2. Scar Appearance and Skin Characteristics

Various studies have utilized the Patient and Observer Scar Assessment Scale (POSAS) to evaluate the outcomes of scar treatments involving autologous fat grafting. This scale is divided into two parts: one part is completed by patients and the other by observers. Each version comprises six items. For observers, these items are vascularity, pigmentation, thickness, relief, and pliability, while patients assess pain, itching, color, stiffness, thickness, and irregularity [101]. Research by Krastev et al. performed a meta-analysis on the scores derived from patients’ evaluations using the POSAS and observed a statistically significant overall improvement across the majority of these categories, with the exception of itching. Notably, the most remarkable improvements were seen in the areas of scar stiffness, followed by improvements in scar color and irregularity [102]. Complementing these findings, a systematic review by Negenborn et al. highlighted significant enhancements in the elasticity, pliability, and relief of scars post-autologous fat grafting [103]. The observed improvements in scar stiffness and pliability, along with the other areas measured by the POSAS, lend credence to the notion that the regenerative process promoted by AFG contributes to superior tissue quality and a reduction in fibrosis [104].

### 3.3. Volume and Scar Contour

Beyond its regenerative capabilities, fat naturally serves as an effective filler, correcting volume deficits and contour irregularities. Consequently, AFG has become a favored approach for rectifying post-surgical deformities, congenital anomalies, and, increasingly, for treating atrophic scars or scars marred by contour disruptions [102,105]. Numerous studies have reported positive results in mitigating the volume loss associated with scar tissues [67,106,107,108]. However, the challenge of volume retention post-AFG has been well-documented, with reported volume reductions reaching up to 70%—a significant hurdle in the process [109,110,111]. Yet, more recent research aligns with observations of volume preservation ranging from 31% to 90% over follow-up periods of 12 to 18 months [1,106,112].

Forecasting the extent of volume loss remains a complex issue, encouraging recent initiatives aimed at enhancing the rate of graft take and its longevity. Researchers have concentrated on strategies to encourage graft revascularization, thereby improving fat graft durability. Techniques such as cell-assisted lipotransfer (CAL), which involves the enrichment of the graft with culture-expanded ADSCs, and the incorporation of platelet-rich plasma (PRP), have been explored [1]. Although CAL has shown promising outcomes, it also presents challenges, including an increased risk of complications, such as infection or uneven texture [113]. These methods aim to support angiogenesis within the transplanted fat through the upregulated secretion of VEGF, enhancing both the survival and functionality of the graft. While AFG stands as a promising intervention for the treatment of atrophic scars or those with contour irregularities, advancing research in this space is crucial for refining predictions regarding graft retention rates and optimizing outcomes [112,114,115].

### 3.4. Fibrosis and Functional Impairment

Fibrosis is a pathological condition, marked by the excessive buildup of fibrous connective tissue in an organ or tissue, typically as a response to injury, inflammation, or exposure to certain pathological stimuli, such as radiotherapy. This over-accumulation can significantly impair the functionality of the affected organ or tissue. AFG has been identified as an effective intervention for mitigating fibrosis and its associated functional limitations in scarred tissues [116].

The mechanical action of needle injection during AFG has been observed to disrupt fibrotic tissue, contributing to the reduction of fibrosis [6]. Beyond this mechanical effect, the beneficial outcomes of AFG in treating fibrosis, particularly following radiotherapy, are largely attributed to the functional properties of ADSCs through previously discussed growth factors and cytokines with angiogenic capabilities. Radiotherapy-induced fibrosis, a delayed adverse effect of radiation treatment, is characterized by the presence of fibro-necrotic tissue, dystrophic fat lobules, and denser, smaller vascular structures accompanied by perivascular fibrosis. AFG targets these alterations by reducing necrotic areas and promoting neoangiogenesis, thereby improving the quality of the skin [10,25]. The positive impact of AFG on fibrosis, particularly in areas around joints where scar contraction can severely restrict function, is supported by studies focusing on functional deficits. This includes limitations in facial movements following burn injuries and reduced joint mobility. The reported improvements in these areas underscore the efficacy of AFG in enhancing functional outcomes [115,117,118].

The immunomodulatory properties ADSCS exhibit are critical in the context of fibrotic disease [119]. By secreting interleukin-10 (IL-10), transforming growth factor-beta (TGF-β), and prostaglandin E2 (PGE2), ADSCs can shift the local immune environment from a pro-inflammatory to an anti-inflammatory state. This modulation helps to attenuate the chronic inflammation that often precedes and exacerbates fibrosis [120]. TGF-β, in particular, plays a dual role: while it is implicated in the pathogenesis of fibrosis, ADSC-derived TGF-β can also promote the conversion of effector T-cells to regulatory T-cells (T_regs_), fostering an anti-inflammatory milieu [121]. Additionally, ADSCs can inhibit the proliferation and function of pro-inflammatory cells, such as macrophages and T-cells, by engaging with their receptors and signaling pathways, thus preventing the excessive deposition of ECM components characteristic of fibrotic tissue [122].

As previously discussed, a critical aspect of ADSCs’ therapeutic impact involves their influence on the ECM [123]. Fibrosis is marked by an aberrant accumulation of ECM components, such as collagen, fibronectin, and elastin, which contributes to tissue stiffness and functional impairment [124]. ADSCs can regulate ECM remodeling through the secretion of matrix metalloproteinases (MMPs) and their inhibitors (TIMPs). MMPs are enzymes that degrade various ECM proteins, facilitating the removal of excessive fibrous deposits. Simultaneously, ADSCs can alter ECM composition towards a more physiological state by modulating fibroblast activity and collagen synthesis, further supporting tissue repair and reducing fibrotic changes [57].

Beyond these secreted factors, direct interactions between ADSCs and resident cell types within the fibrotic tissue play a vital role. ADSCs can differentiate into various cell lineages, potentially replenishing cells lost to fibrotic injury. Furthermore, through direct cell–cell contact and paracrine signaling, ADSCs can influence the behavior of fibroblasts, the primary effector cells in fibrosis, preventing their excessive activation and the resultant pathological ECM deposition [119].

Thus, the anti-fibrotic effects of AFG, via ADSCs, involve a nuanced orchestration of angiogenic promotion, immune modulation, ECM remodeling, and direct cellular effects [125]. These mechanisms collectively contribute to the attenuation of fibrosis and the restoration of normal tissue architecture and function, highlighting the profound potential of ADSCs in regenerative medicine and fibrosis treatment.

### 3.5. Autologous Far Grafting and Pain Reduction

Lipofilling, a process involving the transplantation of autologous fat tissue, has been documented to significantly mitigate, and in some cases, entirely alleviate pain associated with scar tissue [106,115,118,126]. Reports indicate both immediate and sustained pain relief following the procedure. The notable 2017 review by Riyat et al. [6] highlighted an analgesic effect of lipofilling across a broad spectrum of patients, including those with post-mastectomy pain, facial scars, perineal post-surgical scars, burns, and vaginal lacerations, demonstrating a remarkable efficacy in 832 out of 966 cases.

Brain-derived neurotrophic factor (BDNF), a neurotrophin secreted by ADSCs, is instrumental in nerve regeneration. BDNF promotes the survival and growth of neurons, enhancing nerve repair and potentially contributing to the alleviation of neuropathic pain associated with scar tissue [127]. It exerts its effects by binding to the TrkB receptor on nerve cells, activating intracellular signaling pathways such as the PI3K/Akt and MAPK/ERK, which are crucial for neuronal survival, growth, and differentiation [128].

Additionally, TGF-β is implicated in immunomodulation, specifically in the modulation of T-cell activity. It has a profound effect on the immune system, capable of inducing T-cell apoptosis, promoting the differentiation of T_regs_, and suppressing the activation and proliferation of effector T-cells. Through these actions, TGF-β contributes to reducing inflammation and immunological activity within scar tissue, mechanisms that are closely associated with the attenuation of pain [129].

Moreover, the mechanical disruption of fibrotic adhesions during the lipofilling procedure plays a crucial role in pain reduction. This physical intervention can release entrapped nerves within the scar tissue, directly addressing one of the physical causes of scar-associated pain. The alleviation of nerve entrapment not only contributes to immediate pain relief but also to the long-term restoration of sensory function and reduction in neuropathic pain [6]. These elements collectively foster a regenerative microenvironment, promote nerve repair, modulate immune responses, and mechanically liberate entrapped nerves, leading to a significant reduction in scar-associated pain.

In terms of pain relief, ADSCs exert a significant immunomodulatory effect within scar tissue by producing anti-inflammatory cytokines, such as interleukin-10 (IL-10) and transforming growth factor-beta (TGF-β). IL-10 counteracts inflammatory signaling by suppressing the production of pro-inflammatory cytokines, thus potentially dampening the pain signals emanating from the scarred area [130]. TGF-β contributes to this analgesic effect by regulating immune cell functions, including the mitigation of effector T-cell proliferation, and by inducing the development of regulatory T-cells, fostering an immunosuppressive state that can lead to a reduction in nociception [131]. Furthermore, ADSCs promote neural repair and reduce neuropathic pain via the secretion of neurotrophic factors, such as BDNF. BDNF acts through TrkB receptors on nerve cells to activate downstream signaling pathways such as PI3K/Akt and MAPK/ERK, which are vital for nerve cell survival and regeneration, offering a potential mechanism for the alleviation of neuropathic pain often associated with scar tissue [132]. Adding to the discussion on BDNF, the roles of nerve growth factor (NGF) and glial cell line-derived neurotrophic factor (GDNF) secreted by ADSCs are also central to these processes. NGF is crucial for the survival and maintenance of sympathetic and sensory neurons, while GDNF supports the survival of motor neurons and promotes axonal growth [133]. These neurotrophic factors play essential roles in repairing nerve damage associated with scarring, contributing to pain alleviation and the restoration of sensory function.

## 4. Clinical Applications

Our current understanding positions AFG as a pivotal intervention for the management of a wide spectrum of scar types. This efficacy is largely attributable to the dual action of AFG: its ability to restore volume and its regenerative capabilities, the latter of which is enhanced by the presence of ADSCs. These cells provide the graft with properties that exceed volume augmentation, enabling significant improvements in tissue repair and functionality [6].

AFG is particularly beneficial for the treatment of depressed scars or those characterized by volume loss, exploiting the inherent volumetric enhancement offered by the transferred fat to rectify such deficiencies [134]. Furthermore, AFG stands out in its capacity to address fibrosis—a prevalent challenge in scar management. It proves effective across a range of scenarios, from scars that result in functional impairment to chronic wounds and areas of fibrotic tissue sequelae following radiotherapy. The intervention targets the fibrotic tissue directly, promoting remodeling and reducing the pathological accumulation of fibrous connective tissue [135]. Additionally, the analgesic properties of AFG, documented in numerous patient outcomes, underscore its value as a therapeutic option for painful scars. By alleviating pain, AFG not only addresses the physical aspects of scarring but significantly enhances the quality of life for individuals affected by post-surgical pain, burns, or other painful scar conditions.

Beyond these applications, the substantial regenerative potential inherent in autologous fat grafts positions AFG as a promising approach for the treatment of chronic wounds. By fostering an environment conducive to wound healing, AFG can play a crucial role in preventing or minimizing pathological scarring, facilitating the restoration of skin integrity and function. AFG leverages a multifaceted mechanism of action—encompassing volume restoration, regenerative enhancement, fibrosis mitigation, pain relief, and wound healing promotion—to offer a comprehensive solution for scar management [136]. This broad-spectrum efficacy, underpinned by the biological activities of ADSCs, positions AFG as a versatile and highly effective modality in the evolving landscape of regenerative medicine and scar treatment strategies.

## 5. Limitations of Current Studies and Future Directions

Autologous fat grafting shows considerable promise in treating scars, but there are several challenges in current clinical research. One major issue is the limited number of large-scale, randomized controlled trials, which hinders the ability to generalize findings. Additionally, differences in study designs, patient demographics, and treatment protocols make it difficult to draw consistent comparisons. The evaluation of therapeutic outcomes is often complicated by a lack of standardized scar assessment tools. The subjective nature of patient-reported outcomes, such as changes in pain, itchiness, and appearance, can introduce variability and potential bias. This reliance on non-standardized questionnaires and subjective measures can lead to inconsistent results and interpretations. Our study reflects these broader issues: while we provide valuable insights, our conclusions are limited by the available literature and the variability in study methodologies. The findings may not be universally applicable, emphasizing the need for further research with larger, more diverse participant groups and standardized assessment methods to confirm and expand on these initial observations.

Furthermore, enhanced understanding and innovative approaches are crucial to fully harness the potential of AFG. A pivotal area of future investigation involves the cellular and molecular biology of adipocytes ADSCs post-transplantation. Studies should aim to dissect the survival pathways activated in transplanted fat cells and the role of the extracellular matrix in supporting these cells. This includes examining how ADSCs interact with their new environment, particularly focusing on the signaling pathways, such as P13K/Akt, Wnt/β-catenin, and HIPPO, which are crucial for cell survival, proliferation, and differentiation. By understanding these mechanisms, researchers can develop targeted interventions to enhance cell viability, such as genetically modifying ADSCs to overexpress specific survival or angiogenic factors.

The method by which fat is processed before transplantation affects the quality and outcomes of AFG. Future directions should include optimizing centrifugation settings to balance between removing excess oil and free lipid, which can cause inflammation, and preserving the structural integrity of the adipose tissue. Advanced techniques, such as the cryopreservation of adipose tissue, might also be explored for its potential to allow off-the-shelf availability of pre-processed fat, ensuring consistent quality and reducing operative times. 

Further, combining AFG with other regenerative techniques offers a promising route to maximize therapeutic outcomes. For example, the integration of AFG with microneedling could be further explored to determine optimal needling depths and patterns that best enhance graft take. Additionally, leveraging laser therapy pre- or post-AFG could help modulate inflammatory responses or stimulate collagen remodeling, enhancing both the aesthetic and functional outcomes of scar treatment. The adjunct use of PRP with AFG should be studied more extensively to understand its effects on graft survival and angiogenesis. Future studies could investigate the optimal concentration and activation method of PRP that best supports the survival and integration of the fat graft, possibly customizing PRP preparation based on patient-specific factors.

Research into how AFG can be tailored to treat different types of scars, such as atrophic versus hypertrophic scars, could lead to more personalized and effective treatments. This could involve varying the size of the fat graft particles or the depth of placement depending on the scar characteristics and the desired outcome. Additionally, there is a need for more longitudinal studies to track the long-term outcomes and safety of AFG, as well as comparative studies that pit AFG against other scar treatment modalities. These studies could provide valuable data on the efficacy of AFG in various clinical scenarios and help standardize treatment protocols.

The development and application of advanced imaging technologies such as MRI or OCT (Optical Coherence Tomography) to monitor the transplanted fat could significantly enhance the precision of AFG procedures. Real-time monitoring techniques could assist in optimizing the placement of grafts and immediately evaluating the success of the transplantation process. By addressing these key areas, future research can substantially refine the application of AFG, enhance its effectiveness, and expand its use in clinical practice, providing patients with improved outcomes in scar treatment and tissue repair.

## 6. Conclusions

AFG, enhanced by ADSCs, offers a superior approach to scar management by providing both aesthetic and functional benefits. ADSCs improve scarred tissues through angiogenesis, immunomodulation, cellular differentiation, and extracellular matrix remodeling. AFG’s effectiveness spans various scar conditions, including volume loss, fibrosis, and chronic pain, and also aids in healing chronic wounds and reducing pathological scarring.

Advancements in ADSC biology and fat grafting are paving the way for personalized treatments tailored to individual needs, maximizing outcomes and minimizing risks. Future integration of bioengineering, 3D printing, and gene editing with AFG and ADSCs could revolutionize regenerative therapies, enhancing aesthetic improvements and the quality of life for those affected by scars.

## Figures and Tables

**Table 1:** Autologous fat grafting: techniques and clinical implications. This table reports some key points about how autologous fat grafting (AFG) techniques are categorized by adipose particle size into macrofat, microfat, and nanofat, each suited for different applications, ranging from large volume augmentations to fine-detail enhancements. Nanofat, in particular, is noted for its high content of stromal vascular fraction (SVF) and stem cells, crucial for tissue regeneration and repair. SVF cells, including ADSCs, are key for graft integration, survival, and differentiation into mature adipocytes, aiding in both volumetric and regenerative outcomes. AFG is effective in improving scar appearance by enhancing skin quality and reducing fibrosis and functional impairment. ADSCs play a significant role in this process by releasing growth factors and cytokines that promote angiogenesis, reduce inflammation, and modulate immune responses, which can alleviate scar-associated pain and improve tissue elasticity and pliability. Studies show significant improvements in scar characteristics post-fat grafting, with positive impacts on volume restoration, contour correction, and functional recovery.

Techniques and Clinical Implication of Autologous Fat Grafting: Key Points	
Overview and Evolution	Autologous adipose tissue is highly accessible, biocompatible, and minimally traumatic surgically. Techniques evolved to use macrofat, microfat, and nanofat for different clinical purposes.
Macrofat Grafting	Uses particles > 2.4 mm, suitable for large volume augmentations (breasts, buttocks). Requires blunt needles with a 2 mm diameter.
Microfat Grafting	Uses particles between 1.2 to 2.4 mm, ideal for delicate areas (forehead, eyelids, hands). Smaller particles facilitate smoother injections and detailed enhancements.
Nanofat Grafting	Involves mechanical emulsification and filtration of microfat. Contains a high concentration of SVF cells and ADSCs, promoting tissue repair and regeneration
SVF and ADSCs	Vital for graft integration and survival, capable of transforming into mature adipocytes. CD34+ cells within SVF show significant proliferative capabilities and enhance graft viability through angiogenesis.
Centrifugation process	Critical for isolating the SVF, ensuring optimal regenerative potential. Different mechanical and enzymatic methods impact cell yield and viability.
Donor Site Selection	No significant difference in fat quality or graft survival among various sites. Selection should consider patient-specific factors and clinical needs.
Advantages and Limitations	Macrofat is effective for volume but poses embolism risk in superficial tissues. Microfat offers safer and precise enhancements. Nanofat is particularly useful for therapeutic applications such as scar revisions.
Regenerative Properties	SVF and ADSCs significantly improve skin quality and scar appearance. Enhance tissue repair through angiogenesis, immune modulation, and ECM remodeling.
Volume Retention	A major challenge, with reductions of up to 70%. Strategies such as cell-assisted lipotransfer (CAL) and PRP aim to improve graft longevity and retention.
Impact on Fibrosis	AFG reduces fibrosis, improves tissue quality and enhances functional outcomes. ADSCs play a crucial role in mitigating chronic inflammation and promoting angiogenesis.
Pain Reduction	AFG significantly alleviates pain associated with scar tissue. ADSCs secrete neurotrophic factors such as BDNF, promoting nerve repair and reducing neuropathic pain.

## References

[1] Li L., Pan S., Ni B., Lin Y. (2014). Improvement in autologous human fat transplant survival with SVF plus VEGF–PLA nano-sustained release microspheres. Cell Biol. Int..

[2] Gentile P. (2023). Lipofilling Enriched with Adipose-Derived Mesenchymal Stem Cells Improves Soft Tissue Deformities and Reduces Scar Pigmentation: Clinical and Instrumental Evaluation in Plastic Surgery. Aesthetic Plast. Surg..

[3] Jatin B., Karki D., Ahluwalia C., Muthukumar V., Karki D. (2023). Lipofilling—A Regenerative Alternate for Remodeling Burn Scars: A Clinico-Immunohistopathological Study. Indian J. Plast. Surg..

[4] Brayfield C.C., Marra K., Rubin J.P. (2010). Adipose Stem Cells for Soft Tissue Regeneration. Handchir. Mikrochir. Plast. Chir..

[5] Hanson S.E. (2021). The Future of Fat Grafting. Aesthetic Surg. J..

[6] Riyat H., Touil L.L., Briggs M., Shokrollahi K. (2017). Autologous fat grafting for scars, healing and pain: A review. Scars Burn. Health.

[7] Zocchi M.L., Prantl L., Oliinyk D., Knoedler L., Siegmund A., Ahmad N., Duscher D., Larcher L., Raposio E., Pagani A. (2024). Potential benefits of adipose–derived SVF and MSCs to regenerate damaged tissues from alloplastic synthetic materials. Eur. J. Plast. Surg..

[8] Spiekman M., Van Dongen J.A., Willemsen J.C., Hoppe D.L., Van Der Lei B., Harmsen M.C. (2017). The power of fat and its adipose-derived stromal cells: Emerging concepts for fibrotic scar treatment: Adipose tissue and ADSC for fibrotic scar treatment. J. Tissue Eng. Regen. Med..

[9] Stachura A., Paskal W., Pawlik W., Mazurek M.J., Jaworowski J. (2021). The Use of Adipose-Derived Stem Cells (ADSCs) and Stromal Vascular Fraction (SVF) in Skin Scar Treatment—A Systematic Review of Clinical Studies. JCM.

[10] Rigotti G., Charles-de-Sá L., Gontijo-de-Amorim N.F., Takiya C.M., Amable P.R., Borojevic R., Benati D., Bernardi P., Sbarbati A. (2016). Expanded Stem Cells, Stromal-Vascular Fraction, and Platelet-Rich Plasma Enriched Fat: Comparing Results of Different Facial Rejuvenation Approaches in a Clinical Trial. Aesthet. Surg. J..

[11] De Ugarte D.A., Morizono K., Elbarbary A., Alfonso Z., Zuk P.A., Zhu M., Dragoo J.L., Ashjian P., Thomas B., Benhaim P. (2003). Comparison of Multi-Lineage Cells from Human Adipose Tissue and Bone Marrow. Cells Tissues Organs.

[12] Rydén M., Dicker A., Götherström C., Åström G., Tammik C., Arner P., Le Blanc K. (2003). Functional characterization of human mesenchymal stem cell-derived adipocytes. Biochem. Biophys. Res. Commun..

[13] Gimble J.M., Guilak F. (2003). Adipose-derived adult stem cells: Isolation, characterization, and differentiation potential. Cytotherapy.

[14] Rehman J., Traktuev D., Li J., Merfeld-Clauss S., Temm-Grove C.J., Bovenkerk J.E., Pell C.L., Johnstone B.H., Considine R.V., March K.L. (2004). Secretion of Angiogenic and Antiapoptotic Factors by Human Adipose Stromal Cells. Circulation.

[15] Cao Y., Sun Z., Liao L., Meng Y., Han Q., Zhao R.C. (2005). Human adipose tissue-derived stem cells differentiate into endothelial cells in vitro and improve postnatal neovascularization in vivo. Biochem. Biophys. Res. Commun..

[16] Tsuji W. (2014). Adipose-derived stem cells: Implications in tissue regeneration. WJSC.

[17] Chen X., Yan L., Guo Z., Chen Z., Chen Y., Li M., Huang C., Zhang X., Chen L. (2016). Adipose-derived mesenchymal stem cells promote the survival of fat grafts via crosstalk between the Nrf2 and TLR4 pathways. Cell Death Dis..

[18] Liu T., Zhang L., Joo D., Sun S.C. (2017). NF-κB signaling in inflammation. Signal Transduct. Target. Ther..

[19] Ting H.K., Chen C.L., Meng E., Cherng J.-H., Chang S.-J., Kao C.-C., Yang M.-H., Leung F.-S., Wu S.-T. (2021). Inflammatory Regulation by TNF-α-Activated Adipose-Derived Stem Cells in the Human Bladder Cancer Microenvironment. Int. J. Mol. Sci..

[20] Yu H., Pardoll D., Jove R. (2009). STATs in cancer inflammation and immunity: A leading role for STAT3. Nat. Rev. Cancer.

[21] Hutchins A.P., Poulain S., Miranda-Saavedra D. (2012). Genome-wide analysis of STAT3 binding in vivo predicts effectors of the anti-inflammatory response in macrophages. Blood.

[22] Wang Y., Chu Y., Yue B., Ma X., Zhang G., Xiang H., Liu Y., Wang T., Wu X., Chen B. (2017). Adipose-derived mesenchymal stem cells promote osteosarcoma proliferation and metastasis by activating the STAT3 pathway. Oncotarget.

[23] Xia T., Zhang M., Lei W., Yang R., Fu S., Fan Z., Yang Y., Zhang T. (2023). Advances in the role of STAT3 in macrophage polarization. Front. Immunol..

[24] Hutchins A.P., Diez D., Miranda-Saavedra D. (2013). The IL-10/STAT3-mediated anti-inflammatory response: Recent developments and future challenges. Brief. Funct. Genom..

[25] Rigotti G., Marchi A., Galiè M., Baroni G., Benati D., Krampera M., Pasini A., Sbarbati A. (2007). Clinical Treatment of Radiotherapy Tissue Damage by Lipoaspirate Transplant: A Healing Process Mediated by Adipose-Derived Adult Stem Cells. Plast. Reconstr. Surg..

[26] Garza R.M., Paik K.J.A., Chung M.T.B., Duscher D., Gurtner G.C., Longaker M.T.M., Wan D.C. (2014). Studies in Fat Grafting: Part, I.I.I. Fat Grafting Irradiated Tissue—Improved Skin Quality and Decreased Fat Graft Retention. Plast. Reconstr. Surg..

[27] Sultan S.M., Stern C.S., Allen R.J., Thanik V.D., Chang C.C., Nguyen P.D., Canizares O., Szpalski C., Saadeh P.B., Warren S.M. (2011). Human Fat Grafting Alleviates Radiation Skin Damage in a Murine Model. Plast. Reconstr. Surg..

[28] Han T.T.Y., Toutounji S., Amsden B.G., Flynn L.E. (2015). Adipose-derived stromal cells mediate in vivo adipogenesis, angiogenesis and inflammation in decellularized adipose tissue bioscaffolds. Biomaterials.

[29] Shibuya M. (2011). Vascular Endothelial Growth Factor (VEGF) and Its Receptor (VEGFR) Signaling in Angiogenesis: A Crucial Target for Anti- and Pro-Angiogenic Therapies. Genes Cancer.

[30] Murakami M., Simons M. (2008). Fibroblast growth factor regulation of neovascularization. Curr. Opin. Hematol..

[31] Wang Q., Zhou M., Zhang H., Hou Z., Liu D. (2023). Hypoxia Treatment of Adipose Mesenchymal Stem Cells Promotes the Growth of Dermal Papilla Cells via HIF-1α and ERK1/2 Signaling Pathways. Int. J. Mol. Sci..

[32] Takuwa Y. (2010). Roles of sphingosine-1-phosphate signaling in angiogenesis. WJBC.

[33] Yuan K., Jin Y., Lin M.T. (2000). Expression of Tie-2, angiopoietin-1, angiopoietin-2, ephrinB2 and EphB4 in pyogenic granuloma of human gingiva implicates their roles in inflammatory angiogenesis. J. Periodontal Res..

[34] Groppa E., Brkic S., Uccelli A., Wirth G., Korpisalo-Pirinen P., Filippova M., Dasen B., Sacchi V., Muraro M.G., Trani M. (2018). EphrinB2/EphB4 signaling regulates non-sprouting angiogenesis by VEGF. EMBO Rep..

[35] Farooq M., Khan A.W., Kim M.S., Choi S. (2021). The Role of Fibroblast Growth Factor (FGF) Signaling in Tissue Repair and Regeneration. Cells.

[36] Mazini L., Rochette L., Admou B., Amal S., Malka G. (2020). Hopes and Limits of Adipose-Derived Stem Cells (ADSCs) and Mesenchymal Stem Cells (MSCs) in Wound Healing. Int. J. Mol. Sci..

[37] Hu Y., Xiong Y., Zhu Y., Zhou F., Liu X., Chen S., Li Z., Qi S., Chen L. (2023). Copper-Epigallocatechin Gallate Enhances Therapeutic Effects of 3D-Printed Dermal Scaffolds in Mitigating Diabetic Wound Scarring. ACS Appl. Mater. Interfaces.

[38] Du E., Li X., He S., Li X., He S. (2020). The critical role of the interplays of EphrinB2/EphB4 and VEGF in the induction of angiogenesis. Mol. Biol. Rep..

[39] Joussen A.M., Ricci F., Paris L.P., Korn C., Quezada-Ruiz C., Zarbin M. (2021). Angiopoietin/Tie2 signalling and its role in retinal and choroidal vascular diseases: A review of preclinical data. Eye.

[40] Mckinnirey F., Herbert B., Vesey G., McCracken S. (2021). Immune modulation via adipose derived Mesenchymal Stem cells is driven by donor sex in vitro. Sci. Rep..

[41] Sanjabi S., Oh S.A., Li M.O. (2017). Regulation of the Immune Response by TGF-β: From Conception to Autoimmunity and Infection. Cold Spring Harb. Perspect. Biol..

[42] Johnson B.Z., Stevenson A.W., Prêle C.M., Fear M.W., Wood F.M. (2020). The Role of IL-6 in Skin Fibrosis and Cutaneous Wound Healing. Biomedicines.

[43] Li D., Li X., Zhang J., Tang Z., Tian A. (2023). The immunomodulatory effect of IL-4 accelerates bone substitute material-mediated osteogenesis in aged rats via NLRP3 inflammasome inhibition. Front. Immunol..

[44] Qin Y., Ge G., Yang P., Wang L., Qiao Y., Pan G., Yang H., Bai J., Cui W., Geng D. (2023). An Update on Adipose-Derived Stem Cells for Regenerative Medicine: Where Challenge Meets Opportunity. Adv. Sci..

[45] Li X.H., Chen F.L., Shen H.L. (2021). Salidroside promoted osteogenic differentiation of adipose-derived stromal cells through Wnt/β-catenin signaling pathway. J. Orthop. Surg. Res..

[46] Rosen E.D., Sarraf P., Troy A.E., Bradwin G., Moore K., Milstone D.S., Spiegelman B.M., Mortensen R.M. (1999). PPARγ Is Required for the Differentiation of Adipose Tissue In Vivo and In Vitro. Mol. Cell.

[47] Iyer S.S., Cheng G. (2012). Role of Interleukin 10 Transcriptional Regulation in Inflammation and Autoimmune Disease. Crit. Rev. Immunol..

[48] Hu X., Li J., Fu M., Zhao X., Wang W. (2021). The JAK/STAT signaling pathway: From bench to clinic. Signal Transduct. Target. Ther..

[49] Zha K., Tian Y., Panayi A.C., Mi B., Liu G. (2022). Recent Advances in Enhancement Strategies for Osteogenic Differentiation of Mesenchymal Stem Cells in Bone Tissue Engineering. Front. Cell Dev. Biol..

[50] Park H.-S., Ju U.-I., Park J.-W., Song J.Y., Shin D.H., Lee K.-H., Jeong L.S., Yu J., Lee H.W., Cho J.Y. (2016). PPARγ neddylation essential for adipogenesis is a potential target for treating obesity. Cell Death Differ..

[51] Dong L., Li X., Leng W., Guo Z., Cai T., Ji X., Xu C., Zhu Z., Lin J. (2023). Adipose stem cells in tissue regeneration and repair: From bench to bedside. Regen. Ther..

[52] Li C., Wei S., Xu Q., Sun Y., Ning X., Wang Z. (2022). Application of ADSCs and their Exosomes in Scar Prevention. Stem Cell Rev. Rep..

[53] Airuddin S.S., Halim A.S., Wan Sulaiman W.A., Kadir R., Nasir N.A.M. (2021). Adipose-Derived Stem Cell: “Treat or Trick”. Biomedicines.

[54] Lu P., Takai K., Weaver V.M., Werb Z. (2011). Extracellular Matrix Degradation and Remodeling in Development and Disease. Cold Spring Harb. Perspect. Biol..

[55] Lipson K.E., Wong C., Teng Y., Spong S. (2012). CTGF is a central mediator of tissue remodeling and fibrosis and its inhibition can reverse the process of fibrosis. Fibrogenesis Tissue Repair..

[56] Xie C., Mondal D.K., Ulas M., Neill T., Iozzo R.V. (2022). Oncosuppressive roles of decorin through regulation of multiple receptors and diverse signaling pathways. Am. J. Physiol.-Cell Physiol..

[57] Cabral-Pacheco G.A., Garza-Veloz I., la Rosa C.C.-D., Ramirez-Acuña J.M., A Perez-Romero B., Guerrero-Rodriguez J.F., Martinez-Avila N., Martinez-Fierro M.L. (2020). The Roles of Matrix Metalloproteinases and Their Inhibitors in Human Diseases. Int. J. Mol. Sci..

[58] Zhou Z., Chen Y., Chai M., Tao R., Lei Y., Jia Y., Shu J., Ren J., Li G., Wei W. (2019). Adipose extracellular matrix promotes skin wound healing by inducing the differentiation of adipose-derived stem cells into fibroblasts. Int. J. Mol. Med..

[59] Ding P., Lu E., Li G., Sun Y., Yang W., Zhao Z. (2022). Research Progress on Preparation, Mechanism, and Clinical Application of Nanofat. J. Burn. Care Res..

[60] Fontes T., Brandão I., Negrão R., Martins M.J., Monteiro R. (2018). Autologous fat grafting: Harvesting techniques. Ann. Med. Surg..

[61] Tonnard P., Verpaele A., Peeters G., Hamdi M., Cornelissen M., Declercq H. (2013). Nanofat Grafting: Basic Research and Clinical Applications. Plast. Reconstr. Surg..

[62] Fotia C., Massa A., Boriani F., Baldini N., Granchi D. (2015). Hypoxia enhances proliferation and stemness of human adipose-derived mesenchymal stem cells. Cytotechnology.

[63] Eto H., Ishimine H., Kinoshita K., Watanabe-Susaki K., Kato H., Doi K., Kuno S., Kurisaki A., Yoshimura K. (2013). Characterization of Human Adipose Tissue-Resident Hematopoietic Cell Populations Reveals a Novel Macrophage Subpopulation with CD34 Expression and Mesenchymal Multipotency. Stem Cells Dev..

[64] Hsiao S.T., Lokmic Z., Peshavariya H., Abberton K.M., Dusting G.J., Lim S.Y., Dilley R.J. (2013). Hypoxic Conditioning Enhances the Angiogenic Paracrine Activity of Human Adipose-Derived Stem Cells. Stem Cells Dev..

[65] Wu Y., Chen L., Scott P.G., Tredget E.E. (2007). Mesenchymal Stem Cells Enhance Wound Healing Through Differentiation and Angiogenesis. Stem Cells.

[66] Baptista L.S., Do Amaral R.J.F.C., Carias R.B.V., Aniceto M., Claudio-da-Silva C., Borojevic R. (2009). An alternative method for the isolation of mesenchymal stromal cells derived from lipoaspirate samples. Cytotherapy.

[67] Condé-Green A., Baptista L.S., de Amorin N.F.G., de Oliveira E.D., da Silva K.R., Pedrosa C.D.S.G., Borojevic R., Pitanguy I. (2010). Effects of Centrifugation on Cell Composition and Viability of Aspirated Adipose Tissue Processed for Transplantation. Aesthetic Surg. J..

[68] Markarian C.F., Frey G.Z., Silveira M.D., Chem E.M., Milani A.R., Ely P.B., Horn A.P., Nardi N.B., Camassola M. (2014). Isolation of adipose-derived stem cells: A comparison among different methods. Biotechnol. Lett..

[69] Shah F.S., Wu X., Dietrich M., Rood J., Gimble J.M. (2013). A non-enzymatic method for isolating human adipose tissue-derived stromal stem cells. Cytotherapy.

[70] Romanov Y.u.A., Darevskaya A.N., Merzlikina N.V., Buravkova L.B. (2005). Mesenchymal Stem Cells from Human Bone Marrow and Adipose Tissue: Isolation, Characterization, and Differentiation Potentialities. Bull. Exp. Biol. Med..

[71] Raposio E., Caruana G., Bonomini S., Libondi G. (2014). A Novel and Effective Strategy for the Isolation of Adipose-Derived Stem Cells: Minimally Manipulated Adipose-Derived Stem Cells for More Rapid and Safe Stem Cell Therapy. Plast. Reconstr. Surg..

[72] Prantl L., Rennekampff H.O., Giunta R.E., Harder Y., von Heimburg D., Heine N., Herold C., Kneser U., Lampert F., Machens H.G. (2016). Aktuelle Erkenntnisse zur Eigenfett Transplantation anhand der neuen Leitlinie “Autologe Fetttransplantation”. Handchir. Mikrochir. Plast. Chir..

[73] Shauly O., Gould D.J., Ghavami A. (2022). Fat Grafting: Basic Science, Techniques, and Patient Management. Plast. Reconstr. Surg.—Glob. Open.

[74] Gause T.M., Kling R.E., Sivak W.N., Marra K.G., Rubin J.P., Kokai L.E. (2014). Particle size in fat graft retention: A review on the impact of harvesting technique in lipofilling surgical outcomes. Adipocyte.

[75] Zocchi M.L., Vindigni V., Pagani A., Pirro O., Conti G., Sbarbati A., Bassetto F. (2019). Regulatory, ethical, and technical considerations on regenerative technologies and adipose-derived mesenchymal stem cells. Eur. J. Plast. Surg..

[76] Uttamani R., Venkataram A., Venkataram J., Mysore V. (2020). Tumescent anesthesia for dermatosurgical procedures other than liposuction. J. Cutan. Aesthet. Surg..

[77] Zhang Q., Liu L.N., Yong Q., Deng J.C., Cao W.G. (2015). Intralesional injection of adipose-derived stem cells reduces hypertrophic scarring in a rabbit ear model. Stem Cell Res. Ther..

[78] Song J., Xian D., Yang L., Xiong X., Lai R., Zhong J. (2018). Pruritus: Progress toward Pathogenesis and Treatment. BioMed Res. Int..

[79] Zocchi M.L., Pagani A., Kalaaji A. (2022). New Strategies in Regenerative Medicine: The Bio-active Composite Grafts. Plastic and Aesthetic Regenerative Surgery and Fat Grafting.

[80] Rochette L., Mazini L., Malka G., Zeller M., Cottin Y., Vergely C. (2020). The Crosstalk of Adipose-Derived Stem Cells (ADSC), Oxidative Stress, and Inflammation in Protective and Adaptive Responses. Int. J. Mol. Sci..

[81] Zocchi M.L., Facchin F., Pagani A., Bonino C., Sbarbati A., Conti G., Vindigni V., Bassetto F. (2022). New perspectives in regenerative medicine and surgery: The bioactive composite therapies (BACTs). Eur. J. Plast. Surg..

[82] Datsi A., Steinhoff M., Ahmad F., Alam M., Buddenkotte J. (2021). Interleukin-31: The “itchy” cytokine in inflammation and therapy. Allergy.

[83] Hao Z., Qi W., Sun J., Zhou M., Guo N. (2023). Review: Research progress of adipose-derived stem cells in the treatment of chronic wounds. Front. Chem..

[84] Krawczenko A., Klimczak A. (2022). Adipose Tissue-Derived Mesenchymal Stem/Stromal Cells and Their Contribution to Angiogenic Processes in Tissue Regeneration. Int. J. Mol. Sci..

[85] Xu H., Ni Y.Q., Liu Y.S. (2021). Mechanisms of Action of MiRNAs and LncRNAs in Extracellular Vesicle in Atherosclerosis. Front. Cardiovasc. Med..

[86] Ling L., Hou J., Liu D., Tang D., Zhang Y., Zeng Q., Pan H., Fan L. (2022). Important role of the SDF-1/CXCR4 axis in the homing of systemically transplanted human amnion-derived mesenchymal stem cells (hAD-MSCs) to ovaries in rats with chemotherapy-induced premature ovarian insufficiency (POI). Stem Cell Res. Ther..

[87] Zeng Z., Lan T., Wei Y., Wei X. (2022). CCL5/CCR5 axis in human diseases and related treatments. Genes Dis..

[88] Furue M., Furue M. (2021). Interleukin-31 and Pruritic Skin. JCM.

[89] Jia Q., Zhao H., Wang Y., Cen Y., Zhang Z. (2023). Mechanisms and applications of adipose-derived stem cell-extracellular vesicles in the inflammation of wound healing. Front. Immunol..

[90] Xue Z., Liao Y., Li Y. (2024). Effects of microenvironment and biological behavior on the paracrine function of stem cells. Genes Dis..

[91] Rautiainen S., Laaksonen T., Koivuniemi R. (2021). Angiogenic Effects and Crosstalk of Adipose-Derived Mesenchymal Stem/Stromal Cells and Their Extracellular Vesicles with Endothelial Cells. Int. J. Mol. Sci..

[92] Elshaer S.L., Bahram S.H., Rajashekar P., Gangaraju R., El-Remessy A.B. (2021). Modulation of Mesenchymal Stem Cells for Enhanced Therapeutic Utility in Ischemic Vascular Diseases. Int. J. Mol. Sci..

[93] Brett E., Duscher D., Pagani A., Daigeler A., Kolbenschlag J., Hahn M. (2022). Naming the Barriers between Anti-CCR5 Therapy, Breast Cancer and Its Microenvironment. Int. J. Mol. Sci..

[94] Turturici G., Tinnirello R., Sconzo G., Geraci F. (2014). Extracellular membrane vesicles as a mechanism of cell-to-cell communication: Advantages and disadvantages. Am. J. Physiol.-Cell Physiol..

[95] Sen C.K., Ghatak S. (2015). miRNA Control of Tissue Repair and Regeneration. Am. J. Pathol..

[96] Trzyna A., Banaś-Ząbczyk A. (2021). Adipose-Derived Stem Cells Secretome and Its Potential Application in “Stem Cell-Free Therapy”. Biomolecules.

[97] Widgerow A.D., Salibian A.A., Lalezari S., Evans G.R.D. (2013). Neuromodulatory nerve regeneration: Adipose tissue-derived stem cells and neurotrophic mediation in peripheral nerve regeneration. J. Neurosci. Res..

[98] Ohtani N. (2022). The roles and mechanisms of senescence-associated secretory phenotype (SASP): Can it be controlled by senolysis?. Inflamm. Regen..

[99] Juhl A.A., Karlsson P., Damsgaard T.E. (2016). Fat grafting for alleviating persistent pain after breast cancer treatment: A randomized controlled trial. J. Plast. Reconstr. Aesthetic Surg..

[100] Kwon D.G., Kim M.K., Jeon Y.S., Nam Y.C., Park J.S., Ryu D.J. (2022). State of the Art: The Immunomodulatory Role of MSCs for Osteoarthritis. Int. J. Mol. Sci..

[101] Lenzi L., Santos J., Raduan Neto J., Fernandes C., Faloppa F. (2019). The Patient and Observer Scar Assessment Scale: Translation for portuguese language, cultural adaptation, and validation. Int. Wound J..

[102] Krastev T.K., Schop S.J., Hommes J., Piatkowski A., Van Der Hulst R.R.W.J. (2020). Autologous fat transfer to treat fibrosis and scar-related conditions: A systematic review and meta-analysis. J. Plast. Reconstr. Aesthetic Surg..

[103] Negenborn V.L., Groen J.W., Smit J.M., Niessen F.B., Mullender M.G. (2016). The Use of Autologous Fat Grafting for Treatment of Scar Tissue and Scar-Related Conditions: A Systematic Review. Plast. Reconstr. Surg..

[104] Gal S., Ramirez J.I., Maguina P. (2017). Autologous fat grafting does not improve burn scar appearance: A prospective, randomized, double-blinded, placebo-controlled, pilot study. Burns.

[105] Bassetto F., Scarpa C., Vindigni V., Téot L., Mustoe T.A., Middelkoop E., Gauglitz G.G. (2020). Invasive Techniques in Scar Management: Fat Injections. Textbook on Scar Management.

[106] Gentile P., Orlandi A., Scioli M.G., Di Pasquali C., Bocchini I., Curcio C.B., Floris M., Fiaschetti V., Floris R., Cervelli V. (2012). A Comparative Translational Study: The Combined Use of Enhanced Stromal Vascular Fraction and Platelet-Rich Plasma Improves Fat Grafting Maintenance in Breast Reconstruction. Stem Cells Transl. Med..

[107] Arcuri F., Brucoli M., Baragiotta N., Stellin L., Giarda M., Benech A. (2013). The Role of Fat Grafting in the Treatment of Posttraumatic Maxillofacial Deformities. Craniomaxillofacial Trauma Reconstr..

[108] Sterodimas A., De Faria J., Nicaretta B., Boriani F. (2011). Autologous Fat Transplantation Versus Adipose-Derived Stem Cell–Enriched Lipografts: A Study. Aesthetic Surg. J..

[109] Delay E., Garson S., Tousson G., Sinna R. (2009). Fat Injection to the Breast: Technique, Results, and Indications Based on 880 Procedures Over 10 Years. Aesthetic Surg. J..

[110] Leong D., Hutmacher D., Chew F., Lim T. (2005). Viability and adipogenic potential of human adipose tissue processed cell population obtained from pump-assisted and syringe-assisted liposuction. J. Dermatol. Sci..

[111] Nishimura T., Hashimoto H., Nakanishi I., Furukawa M. (2000). Microvascular Angiogenesis and Apoptosis in the Survival of Free Fat Grafts. Laryngoscope.

[112] Cervelli V., Scioli M.G., Gentile P., Doldo E., Bonanno E., Spagnoli L.G., Orlandi A. (2012). Platelet-Rich Plasma Greatly Potentiates Insulin-Induced Adipogenic Differentiation of Human Adipose-Derived Stem Cells Through a Serine/Threonine Kinase Akt-Dependent Mechanism and Promotes Clinical Fat Graft Maintenance. Stem Cells Transl. Med..

[113] Yoshimura K., Sato K., Aoi N., Kurita M., Hirohi T., Harii K. (2008). Cell-Assisted Lipotransfer for Cosmetic Breast Augmentation: Supportive Use of Adipose-Derived Stem/Stromal Cells. Aesth Plast. Surg..

[114] Cervelli V., Gentile P., Scioli M.G., Grimaldi M., Casciani C.U., Spagnoli L.G., Orlandi A. (2009). Application of Platelet-Rich Plasma in Plastic Surgery: Clinical and In Vitro Evaluation. Tissue Eng. Part C Methods.

[115] Klinger M., Caviggioli F., Klinger F.M., Giannasi S., Bandi V., Banzatti B., Forcellini D., Maione L., Catania B., Vinci V. (2013). Autologous Fat Graft in Scar Treatment. J. Craniofacial Surg..

[116] Antar S.A., Ashour N.A., Marawan M.E., Al-Karmalawy A.A. (2023). Fibrosis: Types, Effects, Markers, Mechanisms for Disease Progression, and Its Relation with Oxidative Stress, Immunity, and Inflammation. Int. J. Mol. Sci..

[117] Klinger M., Lisa A., Caviggioli F., Maione L., Murolo M., Vinci V., Klinger F.M. (2015). Autologous Fat Grafting Improves Facial Nerve Function. Case Rep. Surg..

[118] Byrne M., O’Donnell M., Fitzgerald L., Shelley O.P. (2016). Early experience with fat grafting as an adjunct for secondary burn reconstruction in the hand: Technique, hand function assessment and aesthetic outcomes. Burns.

[119] Vanderstichele S., Vranckx J.J. (2022). Anti-fibrotic effect of adipose-derived stem cells on fibrotic scars. WJSC.

[120] Seo Y., Kang M.J., Kim H.S. (2021). Strategies to Potentiate Paracrine Therapeutic Efficacy of Mesenchymal Stem Cells in Inflammatory Diseases. Int. J. Mol. Sci..

[121] Ismaeel A., Kim J.S., Kirk J.S., Smith R.S., Bohannon W.T., Koutakis P. (2019). Role of Transforming Growth Factor-β in Skeletal Muscle Fibrosis: A Review. Int. J. Mol. Sci..

[122] Long C., Wang J., Gan W., Qin X., Yang R., Chen X. (2022). Therapeutic potential of exosomes from adipose-derived stem cells in chronic wound healing. Front. Surg..

[123] Yalçın M.B., Bora E.S., Erdoğan M.A., Çakır A., Erbaş O. (2023). The Effect of Adipose-Derived Mesenchymal Stem Cells on Peripheral Nerve Damage in a Rodent Model. JCM.

[124] Ren L.L., Miao H., Wang Y.N., Liu F., Li P., Zhao Y.Y. (2023). TGF-β as A Master Regulator of Aging-Associated Tissue Fibrosis. Aging Dis..

[125] Li Z.-J., Wang L.-Q., Li Y.-Z., Wang C.-Y., Huang J.-Z., Yu N.-Z., Long X. (2021). Application of adipose-derived stem cells in treating fibrosis. WJSC.

[126] Huang H., Shao L., Chen Y., Tang L., Liu T., Li J., Zhu H. (2022). Synergistic strategy with hyperthermia therapy based immunotherapy and engineered exosomes-liposomes targeted chemotherapy prevents tumor recurrence and metastasis in advanced breast cancer. Bioeng. Transl. Med..

[127] Klinger M., Villani F., Klinger F., Gaetani P., Rodriguez YBaena R., Levi D. (2009). Anatomical Variations of the Occipital Nerves: Implications for the Treatment of Chronic Headaches. Plast. Reconstr. Surg..

[128] Bathina S., Das U.N. (2015). Brain-derived neurotrophic factor and its clinical implications. Arch. Med. Sci..

[129] Dahmani A., Delisle J.S. (2018). TGF-β in T Cell Biology: Implications for Cancer Immunotherapy. Cancers.

[130] Steen E.H., Wang X., Balaji S., Butte M.J., Bollyky P.L., Keswani S.G. (2020). The Role of the Anti-Inflammatory Cytokine Interleukin-10 in Tissue Fibrosis. Adv. Wound Care.

[131] Travis M.A., Sheppard D. (2014). TGF-β Activation and Function in Immunity. Annu. Rev. Immunol..

[132] Li Y., Li F., Qin D., Chen H., Wang J., Wang J., Song S., Wang C., Wang Y., Liu S. (2022). The role of brain derived neurotrophic factor in central nervous system. Front. Aging Neurosci..

[133] Palasz E., Wilkaniec A., Stanaszek L., Andrzejewska A., Adamczyk A. (2023). Glia-Neurotrophic Factor Relationships: Possible Role in Pathobiology of Neuroinflammation-Related Brain Disorders. Int. J. Mol. Sci..

[134] Le J.M., Bosworth J.W., Honeywell B., Ananthasekar S., Collawn S.S. (2021). Adipose Grafting for Volume and Scar Release. Ann. Plast. Surg..

[135] El Ayadi A., Jay J.W., Prasai A. (2020). Current Approaches Targeting the Wound Healing Phases to Attenuate Fibrosis and Scarring. Int. J. Mol. Sci..

[136] Brown J.C., Shang H., Yang N., Pierson J., Ratliff C.R.P., Prince N., Roney N., Chan R., Hatem V., Gittleman H. (2020). Autologous Fat Transfer for Scar Prevention and Remodeling: A Randomized, Blinded, Placebo-controlled Trial. Plast. Reconstr. Surg.—Glob. Open.

